# Microalgal Exosome-like Nanovesicles from *Nannochloropsis oculata* Attenuate Melanogenesis Through Tyrosinase Inhibition in B16-F10 Melanoma Cells

**DOI:** 10.3390/md24030107

**Published:** 2026-03-12

**Authors:** Liangquan Xie, Chaoxuan Wu, Weilin Du, Jiaying Chen, Zijie He, Tingting Li, Chuangye Yang, Yuewen Deng, Zhe Zheng

**Affiliations:** 1 Fishery College, Guangdong Ocean University, Zhanjiang 524088, China; 2 Pearl Breeding and Processing Engineering Technology Research Centre of Guangdong Province, Zhanjiang 524088, China; 3 Guangdong Science and Innovation Center for Pearl Culture, Zhanjiang 524088, China; 4 Guangdong Provincial Engineering Laboratory for Mariculture Organism Breeding, Zhanjiang 524088, China; 5 Guangdong Provincial Key Laboratory of Aquatic Animal Disease Control and Healthy Culture, Zhanjiang 524088, China

**Keywords:** exosome-like nanovesicles, microalgae, skin-whitening, melanin, *Nannochloropsis oculata*

## Abstract

As primary producers in aquatic ecosystems, microalgae function not only as a natural source of nourishment for several economically important aquatic species but also as reservoirs of bioactive molecules. Microalgae can secrete exosome-like nanoparticles that transport functional biomolecules, such as proteins and nucleic acids, into the extracellular milieu, thereby mediating intercellular signaling and eliciting ecological or biomedical responses. Although plant-derived exosome-like nanoparticles have attracted attention for their utility in drug delivery and dermatology, the functional properties of microalgae-derived nanoparticles—particularly from species extensively applied in aquaculture—remain inadequately characterized. In this study, exosome-like nanovesicles were isolated from *Nannochloropsis oculata* (N-ELNs), a microalgal species widely used in aquaculture, and their skin-whitening potential was evaluated using the B16-F10 mouse melanoma cell model. The highest N-ELN yield was observed during the adaptation, exponential, and stationary growth phases. Uptake analyses confirmed the efficient internalization of N-ELNs by B16-F10 cells. Cell counting kit-8 assays indicated that N-ELNs exhibited no cytotoxic effects on melanoma cells or normal human dermal fibroblasts (HFF-1). Scratch wound healing assays revealed that N-ELNs exerted no significant effect on cellular migration. In B16-F10 cells, N-ELNs suppressed tyrosinase activity by downregulating *Mitf* and its downstream genes *Tyr* and *Tyrp1*, resulting in a substantial reduction in melanin synthesis (*p* < 0.05). The inhibitory effects of N-ELNs on melanin production, tyrosinase activity, and gene expression of *Tyr*, *Tyrp1*, and *Mitf* were comparable to those of the positive control, arbutin. Collectively, these findings suggest that *N. oculata* exhibits promising skin-whitening properties, providing a novel perspective for clinical applications and supporting the high-value utilization of the microalgae aquaculture industry.

## 1. Introduction

Exosome-like nanovesicles are membrane-enclosed, lipid-bilayer structures produced by cells into the extracellular environment [1]. These vesicles have garnered considerable attention in biomedical research, therapeutic development, and medical esthetics. In animal systems, these nanoscale vesicles transport proteins, nucleic acids, lipids, and other bioactive constituents, facilitating precise intercellular signaling and modulating diverse physiological and pathological processes [2]. Increasing evidence indicates that plant cells secrete exosome-like nanovesicles that contain bioactive compounds with versatile functional properties [3]. Plant-derived exosome-like nanovesicles (PDENs) are advantageous because of their high abundance, biocompatibility, and biodegradability, making them promising candidates for cell-free therapeutic applications [4]. The bilipid membrane structure of PDENs enables recognition and uptake by mammalian cells, allowing cross-kingdom delivery [5]. Their nanoscale size will enable them to penetrate biological barriers, such as the intestinal lining, thereby facilitating the targeted transport of biomolecules—including proteins, lipids, messenger RNA, and microRNA—to recipient cells [6]. Furthermore, PDENs display exceptional structural resilience under diverse pH conditions and thermal ranges, making them suitable for multiple administration modalities, including oral delivery [7].

Approximately 15% of the global population uses skin-lightening products, with Asia representing the largest consumer market [8]. The worldwide market for these products has rapidly expanded, attaining an estimated value of USD 10.8 billion, thereby highlighting their substantial commercial importance [9]. In recent years, PDENs have attracted increasing interest in dermatology, particularly for their potential applications in skin-whitening [4]. Owing to their efficient molecular cargo delivery and high biocompatibility, PDENs are emerging as promising platforms for innovative therapeutic strategies [10]. For instance, *Dendrobium officinale*-derived nanovesicles can accelerate cutaneous wound repair by modulating inflammatory responses and enhancing angiogenesis [11]. Similarly, ginger-derived exosome-like nanovesicles (GDENs) display diverse biomedical activities, including anticancer and anti-inflammatory effects [12]. These nanovesicles possess notable advantages, including efficient cellular uptake, reduced immunogenicity and toxicity, stability in the gastrointestinal tract, and the capacity to traverse the blood–brain barrier, making them attractive candidates for targeted drug delivery and oncological therapy [13]. Substantial evidence supports the role of PDENs as delivery platforms [14]. Resveratrol, a compound with limited bioavailability, has been successfully encapsulated into exosomes derived from *Leontopodium alpinum* (Edelweiss), resulting in synergistic and improved anti-aging effects [15]. Grapefruit-derived PENs have been successfully encapsulated with chemotherapeutic agents and demonstrated targeted antitumor efficacy in experimental models [16].

Microalgae are unicellular photosynthetic microorganisms that efficiently convert solar energy, carbon dioxide, and nutrients into cellular biomass, serving as essential inputs for aquaculture nutrition and as renewable substrates for biodiesel production. However, large quantities of residual byproducts produced during microalgal cultivation are frequently overlooked [17,18,19,20]. Extracellular vesicles (EVs) originating from microalgal cells are discharged into surrounding aquatic matrices [21] and can be effectively isolated from culture media. Exosome-like vesicles have been comprehensively isolated and characterized from the spent culture medium of the marine microalga *Tetraselmis chuii* [22]. Božič reported the successful extraction of membrane-bound nanovesicles from the conditioned media of *Dunaliella tertiolecta* and *Phaeodactylum tricornutum* using differential ultracentrifugation [23]. Despite these developments, the biological roles, structural attributes, and biogenetic origin of microalgae-derived exosome-like nanovesicles remain insufficiently defined, with current insights largely derived from exploratory studies.

Microalgae-derived exosome-like nanovesicles are important for treating skin-related conditions, including skin-whitening and anti-aging. For instance, exosomes isolated from Codium fragile and *Sargassum fusiforme* downregulate the expression of key melanogenesis-related proteins and decrease melanin production in human melanoma cells (MNT-1) and three-dimensional human epidermal models [24]. Extracellular vesicles derived from *Spirulina platensis* (SP-EVs) have been developed to transport bioactive molecules, such as astaxanthin, which displays potent anti-inflammatory properties and promotes tissue regeneration [25]. Exosomes isolated from *Schizochytrium limacinum* have been shown to suppress melanogenesis in animal models markedly [26].

This study evaluated the bioactivity of the marine microalga *N. oculata* in terms of its skin-whitening potential. Although recent studies have confirmed that extracellular vesicles from species such as *Spirulina platensis* and *Schizochytrium limacinum* have anti-melanogenesis potential, these organisms are primarily cultivated for the production of high-value nutritional extracts. In contrast, *N. oculata* is widely used as live feed in aquaculture due to its rapid proliferation [27], but this also generates a large amount of waste culture medium—a residual byproduct that is usually discarded—yet represents a sustainable and economically feasible source for large-scale isolation of nanovesicles. In addition, its defatted biomass is rich in peptides and polysaccharides and exhibits a range of biofunctional properties, including antioxidant and anti-inflammatory activities [28]. *N. oculata* is recognized as a promising and sustainable biological resource with an abundant accumulation of valuable metabolites, including pigments and polyunsaturated fatty acids [29]. The present work emphasizes the development of an efficient protocol for isolating and purifying exosome-like nanovesicles from the culture supernatant of *N. oculata.* It systematically examines their efficacy in attenuating melanogenesis and elucidating the underlying mechanisms. These findings provide novel strategies for the high-value exploitation of *N. oculata* and serve as a source of new bioactive compounds for skin-whitening applications.

## 2. Results

### 2.1. Isolation and Characterization of N-ELNs from N. oculata

**Figure 1:**
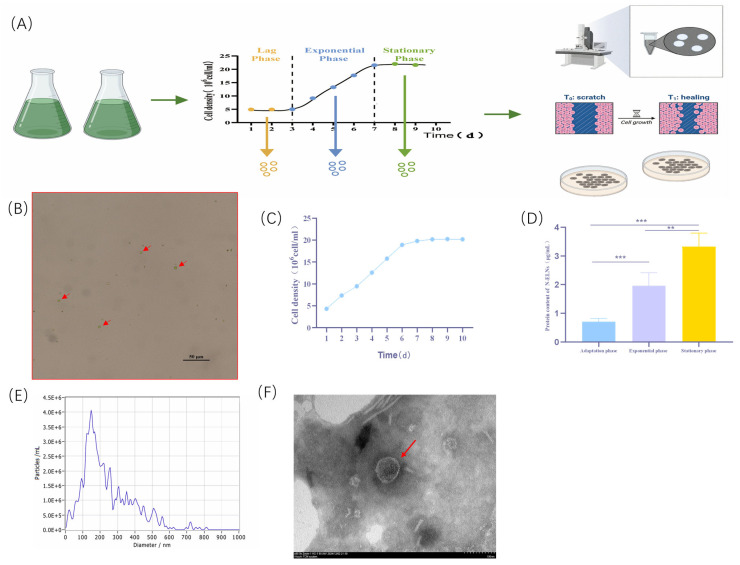
Isolation and characterization of N-ELNs: (**A**) The isolation process of N-ELNs. (**B**) *N. oculata*. (**C**) The cell density of *N. oculata*. (**D**) The protein concentration of N-ELNs isolated at distinct growth phases. Significant differences are indicated by ** *p* < 0.01, and *** *p* < 0.001. (**E**) Size distribution by NTA. (**F**) Representative TEM image (scale bar: 100 nm). Red arrows indicate typical cup-shaped exosome-like nanovesicles.

The relationship between the *N. oculata* growth phase and N-ELN yield was systematically investigated. As illustrated in Figure 1C,D, the microalgal culture reached the stationary phase on day 6 after inoculation. The quantification of N-ELNs in the corresponding culture supernatants using the bicinchoninic acid assay showed protein concentrations of 0.800 μg/mL during the inoculation phase, 1.280 μg/mL in the exponential phase, and 3.832 μg/mL in the stationary phase. The stationary phase yield was significantly elevated compared with earlier stages (*p* < 0.05), indicating that intercellular signaling may intensify during this period, thereby facilitating exosome secretion. N-ELNs obtained from the stationary phase were further characterized. The vesicle diameters were predominantly between 50 and 250 nm, with a mean diameter of 148 nm and a particle concentration of 1.5 × 10^7^ particles/mL (Figure 1E). TEM revealed a classic cup-shaped vesicular morphology (Figure 1F). These physicochemical features are consistent with recognized exosomal characteristics, confirming the successful isolation of N-ELNs with canonical exosome-like properties from the microalgal culture supernatant.

### 2.2. Effect of N-ELNs on B16-F10 Cell and HFF-1 Cell Viability

**Figure 2:**
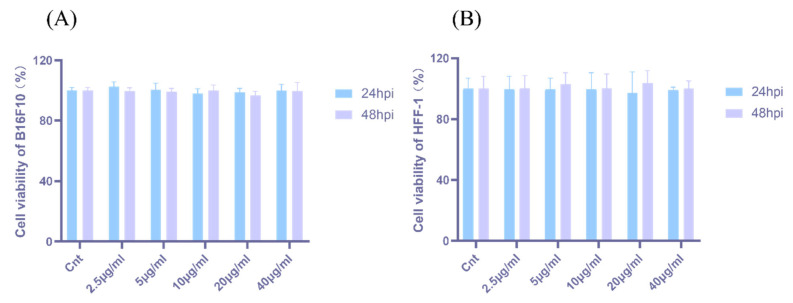
Cytotoxicity of N-ELNs. Cell viability of (**A**) B16F10 and (**B**) HFF-1 cells after treatment with N-ELNs. Data are mean ± SD (*n* = 3). No significant differences (*p* > 0.05).

The biocompatibility of N-ELNs in B16-F10 melanoma cells and HFF-1 cells was evaluated using the CCK-8 assay. This assay is based on the enzymatic reduction of WST-8 to a water-soluble formazan product by intracellular dehydrogenases, with the absorbance correlated with the number of viable cells. As illustrated in Figure 2, exposure to N-ELNs at 2.5, 5, 10, 20, or 40 μg/mL for 24 h or 48 h did not induce a significant decrease in cell viability relative to untreated controls (*p* > 0.05). These findings indicate that N-ELNs exhibit excellent cellular compatibility within the tested concentration range, offering a safe basis for downstream functional investigations.

### 2.3. Cellular Uptake of N-ELNs by B16-F10 Cells

**Figure 3:**
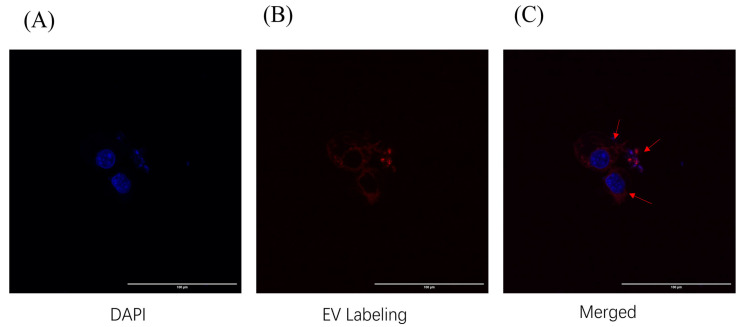
N-ELN uptake by B16-F10 cells. B16-F10 cells were incubated with stained N-ELNs for 12 h. The cells were observed using a confocal laser scanning microscope. N-ELNs: exosome-like nanoparticles from *N. oculata*. (**A**) Cell nuclei stained with DAPI (blue). (**B**) N-ELNs labeled with ExoGlow™ membrane labeling kit (red). (**C**) Merged image of (**A**,**B**). Red arrows indicate internalized N-ELNs within the cytoplasm.

N-ELNs were fluorescently labeled using the ExoGlow™ membrane labeling kit (System Biosciences, Palo Alto, CA, USA), and their internalization by B16-F10 cells was evaluated via confocal laser scanning microscopy. As shown in Figure 3, distinct fluorescent signals were observed within the cytoplasm of B16-F10 cells after 12 h of co-incubation with N-ELNs. In contrast, the control cells treated with the free dye and processed identically exhibited no detectable fluorescence. These results confirm that B16-F10 cells efficiently take up N-ELNs, providing a critical foundation for modulating the intracellular melanin synthesis pathway.

### 2.4. Effect of N-ELNs on B16-F10 Cell Migration

**Figure 4:**
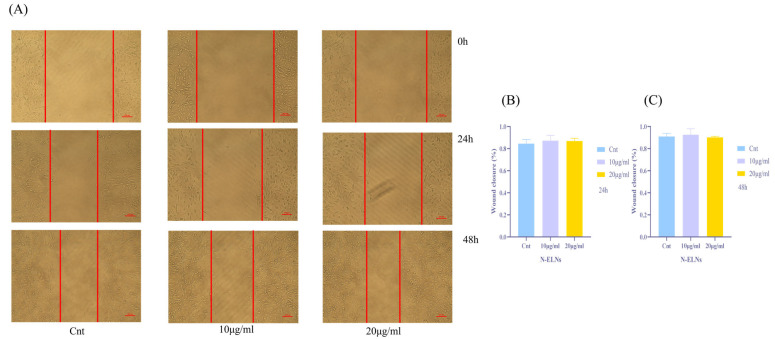
Effect of N-ELNs on the migration of B16-F10 cells: (**A**) representative images of wound healing assay of B16-F10 cells treated with 0, 10, and 20 μg/mL N-ELNs for 24 h and 48 h; (**B**) ratio of cell migration area at 24 h; (**C**) ratio of cell migration area at 48 h. Data are mean ± SD (*n* = 3).

A wound healing assay was conducted to assess the impact of N-ELNs on the migratory behavior of melanoma cells. After creating a scratch, cells were treated with 0, 10, or 20 μg/mL N-ELNs, and images were captured at 0, 24, and 48 h, respectively (Figure 4A). The extent of wound closure was quantified using ImageJ software (version 1.54p, National Institutes of Health, Bethesda, MD, USA). Wound closure rates were comparable across all treatment groups and the control group (0 μg/mL) at both 24 and 48 h (Figure 4B,C), indicating that N-ELNs would not influence the lateral migratory capacity of B16-F10 cells.

### 2.5. N-ELNs Inhibit Melanin Synthesis and Tyrosinase Activity in B16-F10 Cells

**Figure 5:**
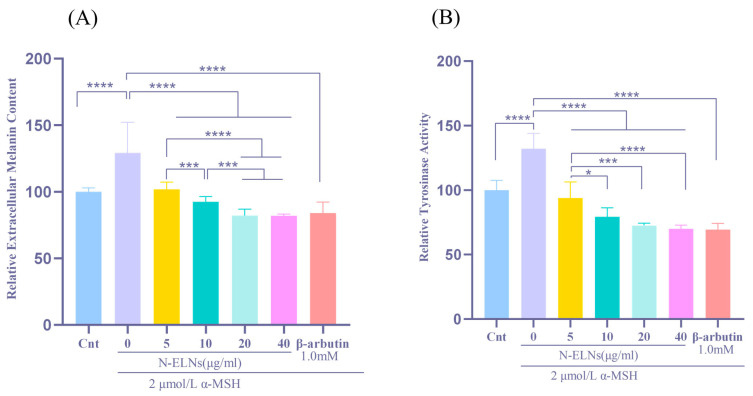
Anti-melanogenic effects of N-ELNs on α-MSH-stimulated B16F10 cells: (**A**) Relative melanin content and (**B**) relative tyrosinase activity in B16F10 cells treated with α-MSH and different concentrations of N-ELNs. Data are presented as mean values. Significant differences are indicated by * *p* < 0.05, *** *p* < 0.001, and **** *p* < 0.0001.

B16-F10 cells were induced with α-MSH to trigger melanogenesis, and extracellular melanin levels were quantified to assess the anti-melanogenic effect of N-ELNs. As depicted in Figure 5A, α-MSH stimulation markedly enhanced melanin synthesis relative to the untreated control (*p* < 0.0001). Co-treatment with 20 and 40 μg/mL N-ELNs resulted in a pronounced concentration-dependent decrease in melanin content (*p* < 0.05, *p* < 0.01, and *p* < 0.0001, respectively), demonstrating the efficacy of N-ELNs in counteracting α-MSH-induced melanogenesis in B16-F10 cells.

Intracellular enzymatic activity was measured using an L-DOPA (Sigma-Aldrich, St. Louis, MO, USA) conversion assay, and absorbance was monitored at 475 nm to determine whether this anti-melanogenic effect is associated with the modulation of the rate-limiting enzyme tyrosinase. As illustrated in Figure 5B, α-MSH substantially elevated tyrosinase activity (*p* < 0.0001), whereas treatment with 20 and 40 μg/mL N-ELNs significantly inhibited enzyme activity (*p* < 0.01 and *p* < 0.0001, respectively). These findings indicate that N-ELNs can partially suppress melanin synthesis by downregulating tyrosinase activity.

### 2.6. Effect of N-ELNs on Melanogenesis-Related Gene Expression

**Figure 6:**
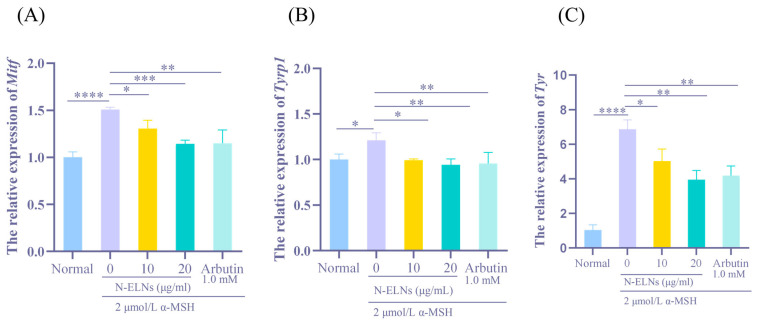
Effect of N-ELNs on mRNA levels of melanogenesis-related factors in B16-F10 cells. B16-F10 cells were incubated with N-ELNs (10 or 20 μg/mL) and α-MSH (0.20 μM). After 48 h, the mRNA expression levels of (**A**) *Mitf*, (**B**) *Tyrp1*, and (**C**) *Tyr* were analyzed. Significant differences are indicated by * *p* < 0.05, ** *p* < 0.01, *** *p* < 0.001, and **** *p* < 0.0001.

The mRNA expression of melanogenesis-related genes was quantified using RT-qPCR to investigate the transcriptional mechanism underlying the anti-melanogenic effect of N-ELNs. As shown in Figure 6, α-MSH exposure can upregulate *Mitf*, *Tyr*, and *Tyrp1* expression, whereas N-ELN treatment will significantly reverse these effects. Specifically, relative to the α-MSH-stimulated model, N-ELNs can strongly suppress the expression levels of *Mitf* (*p* < 0.01, Figure 6A), *Tyrp1* (*p* < 0.01, Figure 6B), and *Tyr* (*p* < 0.01, Figure 6C). These findings indicate that N-ELNs likely suppress Mitf, leading to downregulation of tyrosinase family genes and a coordinated reduction in melanin synthesis.

## 3. Discussion

This study comprehensively evaluated the anti-melanogenic properties of N-ELNs derived from the marine microalga *N. oculata*. N-ELNs were successfully isolated from the culture supernatant and demonstrated dual functionality as both a natural bioactive compound and a potential nanocarrier for skin-whitening applications. These findings offer a novel approach and robust experimental evidence to support the development of next-generation, highly efficient, safe, and sustainable pigmentation-control and functional cosmetic formulations.

The growth cycle of *N. oculata* can be categorized into four characteristic phases [30]. In this study, the isolated N-ELNs displayed canonical features of exosome-like nanovesicles, including a distinct cup-shaped structure and a particle size range of 50–250 nm, with an average diameter of 148 nm. For comparison, PDENs isolated from the leaves of the model plant Arabidopsis thaliana also exhibit a unique cup-shaped morphology under TEM, with a reported mean diameter of approximately 266 nm [31]. Similarly, PDENs obtained from potato roots and peels display comparable spherical morphology with size distributions in the range of 132–165 nm [32]. *Haematococcus pluvialis*-derived extracellular vesicles (HpEVs) secreted under stress conditions across different growth phases show typical exosomal biophysical characteristics, including spherical or quasi-spherical shapes enclosed by a bilayer membrane and consistent cup-shaped morphology across phases [33]. Beyond characterizing their biophysical properties, the biochemical characteristics of exosome-like vesicles are equally crucial for understanding their functional mechanisms and establishing identification criteria. In recent years, research on exosome-like vesicles derived from plants and microalgae has progressively established identification frameworks for protein markers in these organisms. In plants, studies have proposed several candidate marker protein families, including aquaporins, subunits of the vacuolar ATPase complex, bundle-like arabinogalactan proteins, tetradienyl–tetrameric proteins (e.g., TET8, TET9), synaptophysin-like fusion proteins (e.g., PEN1), germinin-like proteins, and calreticulin. Notably, these studies confirm that classical mammalian exosome markers (CD9, CD63, CD81) exhibit extremely low conservation in plants [34]. Research on exosome-like vesicles in microalgae remains relatively scarce, though existing studies suggest that certain proteins involved in vesicle transport and stress responses—such as Rab GTPases (Rab5, Rab7, and Rab11) and heat shock proteins (HSP70, HSP90)—may exhibit greater conservation across different microalgal species. For instance, Rab proteins, as key regulators of vesicle transport, have been consistently validated in exosome-like vesicles from *H. pluvialis* [35] and *Chlamydomonas reinhardtii* [36]. However, intriguingly, recent antibody detection studies have identified the presence of CD9, CD63, and CD81 in *Sargassum fulvellum*, belonging to the phylum Phaeophyta [37]. This discrepancy may reflect both fundamental differences arising from evolutionary divergence between algae and the potential species- or phylum-specific nature of exosome-like vesicle protein markers, particularly when comparing across algal phyla. Consequently, while Rab GTPases and heat shock proteins may serve as relatively conserved functional components within microalgal exosome-like vesicles, establishing reliable and standardized identification markers for microalgal exosome-like vesicles necessitates broader cross-species comparative studies. Regarding the N-ELNs derived from *N. oculata* examined in this study, their biochemical properties remain poorly characterized. This study has confirmed that N-ELNs exhibit physical characteristics (morphology, particle size, electron microscopy) consistent with typical vesicles. However, further investigation combining proteomics, lipidomics, and metabolomics is required to elucidate their biochemical properties. This will contribute towards establishing clearer identification criteria within the field of microalgal exosome vesicle research.

N-ELNs isolated from *N. oculata* cultures during the stationary growth phase exhibited the highest yield. Several biological factors may explain the observed relationship between microalgal growth dynamics and N-ELN production. First, the elevated cell density characteristic of the stationary phase provides a greater number of vesicle-producing cells, which likely contributes to the increased yield. During this phase, intensified competition for nutrients and space suppresses cellular proliferation and may stimulate the release of inhibitory or stress-related factors [38]. Exosomes function as carriers of diverse signaling molecules and mediate intercellular communication under environmental stress, thereby coordinating population-level adaptive responses [39]. Accordingly, it is proposed that heightened intraspecific competition during the stationary phase of *N. oculata* promotes increased N-ELN secretion and enhances its biological activity through the selective packaging of bioactive cargos, including proteins, RNAs, and lipids, which may confer competitive advantages. *H. pluvialis* vesicles released during early growth stages were enriched in proteins associated with primary metabolism, cell division, and energy production, whereas vesicles derived from later growth stages preferentially contained proteins involved in cell wall biosynthesis and secondary metabolism [33]. These findings indicate that cells actively and selectively sort specific proteins into secreted exosomes in response to distinct physiological states and stress conditions [36]. However, the molecular composition of N-ELNs has not yet been identified or characterized, so it remains to be explored.

The present study aimed to evaluate the biological functions of N-ELNs, particularly their potential roles in skin disease. Skin-whitening and anti-aging remain major concerns in the management of skin-related conditions, particularly in Asian populations [8]. PDENs have attracted increasing interest in dermatological applications through two principal mechanisms: functioning as delivery vehicles for therapeutic agents or acting directly as bioactive treatment modalities [40]. Therefore, a systematic evaluation of cellular uptake and cytotoxicity is necessary. The murine melanoma cell line B16F10 was employed as a well-established model for investigating melanogenesis and assessing the bioactivity of vesicles derived from plants and microalgae [41]. Fluorescent labeling experiments confirmed that B16-F10 cells efficiently internalized N-ELNs within 12 h. The CCK-8 assay, which assesses cellular metabolic activity as an indicator of viability, was used to evaluate cytotoxicity [42]. The HFF-1 cell line, a standard human dermal fibroblast model, was used to examine the overall biocompatibility and potential cytotoxic effects of N-ELNs on non-malignant skin cells [43]. After 48 h of treatment, N-ELN concentrations of up to 40 μg/mL did not significantly reduce cell viability in either B16-F10 or HFF-1 cells. To further determine whether N-ELNs interfere with fundamental physiological functions, a scratch wound healing assay was conducted. At a non-toxic concentration (20 μg/mL), N-ELNs did not impair cell migration or wound closure in either B16-F10 or HFF-1 cells. Collectively, these findings indicate that N-ELNs exhibit favorable cellular safety profiles and do not adversely affect cellular repair processes.

Skin pigmentation is a complex physiological mechanism regulated by melanocytes, with photoprotection as its primary biological function [44]. Dysregulation of this process, however, underlies pathological hyperpigmentation disorders such as melasma, making pigmentation control not only a cosmetic objective but also a clinically relevant concern [45]. Melanin synthesis suppression through the inhibition of the rate-limiting enzyme tyrosinase remains the central strategy in the development of skin-lightening agents [46]. Arbutin, one of the most widely investigated skin-whitening agents, primarily acts as a competitive inhibitor of tyrosinase, directly attenuating enzymatic activity and reducing melanin synthesis [47]. Nevertheless, prolonged or high-dose arbutin application has been associated with skin irritation and contact dermatitis [48]. Furthermore, the limitations related to its chemical stability and transdermal delivery continue to restrict its broad application. In the present study, an α-MSH-induced melanogenesis model was established to evaluate the anti-melanogenic effects of N-ELNs. N-ELNs were first confirmed to suppress tyrosinase activity in B16-F10 cells and subsequently demonstrated a dose-dependent inhibition of α-MSH-induced melanogenesis. At a concentration of 30 μg/mL, the inhibitory efficacy of N-ELNs showed no statistically significant difference compared with the positive control group treated with 1 mM arbutin, indicating that N-ELNs exhibit anti-melanogenic activity comparable to that of established skin-whitening agents.

Tyrosinase is a rate-limiting enzyme in the melanogenesis pathway and a pivotal target for assessing the efficacy of skin-lightening [49]. Tyrosinase activity may be suppressed by two principal mechanisms: direct inhibition of the enzymatic protein or downregulation of its expression at the transcriptional or translational level [50]. To elucidate the underlying mechanism, the transcriptional expression of the key melanogenic genes *Tyr* and *Tyrp1* was examined. N-ELN treatment substantially downregulated *Tyr* and *Tyrp1* mRNA levels, with a magnitude of suppression comparable to that observed in the arbutin-treated group. *Tyr* and *Tyrp1* expression is transcriptionally regulated by the microphthalmia-associated transcription factor Mitf [51]. Under α-MSH stimulation, binding of α-MSH to the melanocortin-1 receptor (MC1R) on melanocytes activates adenylate cyclase, leading to increased intracellular cAMP levels and subsequent induction of *Mitf* transcription via the PKA–CREB signaling pathway [52]. Mitf functions as a master transcriptional regulator by binding to M-box and E-box elements within the promoters of *Tyr*, *Tyrp1*, and other melanogenesis-related genes. N-ELNs significantly downregulated *Mitf* mRNA expression, indicating that N-ELNs may attenuate α-MSH-induced melanogenesis by inhibiting Mitf-mediated transcriptional activation [53]. N-ELNs display concentration-dependent inhibitory effects on *Mitf*, *Tyrp1*, and *Tyr* expression, with the 20 μg/mL treatment group generally demonstrating stronger inhibition than the 10 μg/mL group, indicating a clear dose-response relationship. The inhibitory efficacy of N-ELNs was comparable to that of arbutin and exceeded it in some parameters. These results indicate that N-ELNs may represent a promising novel skin-whitening bioactive agent with considerable developmental potential, meriting further investigation into their specific molecular targets and mechanistic pathways.

## 4. Materials and Methods

### 4.1. Culture of N. oculata

The marine microalga *N. oculata* (provided by the Algae Laboratory of Guangdong Ocean University, Zhanjiang, China) was inoculated into Erlenmeyer flasks at a ratio of 1:50 and supplemented with 107-13 culture medium (Shanghai Guangyu Biotechnology, Shanghai, China) during the exponential phase. Seawater collected from Zhanjiang (21°16′12″ N, 110°21′27″ E) was used as the culture medium base. Cultivation was performed at 25 ± 1 °C with a salinity of 30 ± 2 psu under continuous illumination combining natural light and 70 W fluorescent lamps. Cultures were continuously aerated throughout the incubation period. Harvesting was conducted when the algal density reached 2.175 × 10^7^ cells/mL.

### 4.2. Extraction of N-ELNs

N-ELN isolation was performed under refrigerated conditions to maintain vesicle integrity. First, 50 mL of the culture was centrifuged at 4000× *g* for 10 min to remove the cells. The supernatant was then centrifuged at 10,000× *g* for 40 min to eliminate debris, followed by sequential filtration via 0.45 μm and 0.22 μm membrane filters. The filtrate was concentrated to 250 μL using a 30 kDa MWCO ultrafiltration tube at 5000× *g* for 20 min. Subsequently, 63 μL ExoQuick reagent (System Biosciences, Palo Alto, CA, USA) was added, incubated at 4 °C for 8 h, and centrifuged at 1500× *g* for 5 min. The resulting pellet was resuspended in 100 μL of PBS for downstream analyses. Protein concentration of N-ELNs was determined using the Bicinchoninic Acid (BCA) assay kit (Beyotime, P0012, Shanghai, China). Briefly, bovine serum albumin (BSA) standards (0, 0.025, 0.05, 0.1, 0.2, 0.3, 0.4, and 0.5 mg/mL) were prepared in PBS. N-ELN samples were diluted appropriately to fall within the linear range of the standard curve. Samples and standards were incubated with BCA working reagent at 37 °C for 30 min, and absorbance was measured at 562 nm. A standard curve was generated (linear regression, R^2^ > 0.99) and used to calculate sample concentrations.

### 4.3. Cell Culture

Mouse melanoma B16-F10 cells (Promega Corporation, CL-0319, Madison, WI, USA) and human foreskin fibroblast HFF-1 cells (SERANA, S-H0123, Pessin, Germany) were maintained in Dulbecco’s modified Eagle’s medium (DMEM; Promega Corporation, PM150210) supplemented with 10% fetal bovine serum (FBS; Promega Corporation, 164220, Madison, WI, USA) and 1% penicillin–streptomycin (Promega Corporation, PB180120, Madison, WI, USA) at 37 °C in a humidified incubator containing 5% CO_2_. Cells were subcultured using trypsin-EDTA upon reaching 80–90% confluence and prepared for subsequent experiments.

### 4.4. Nanoparticle Tracking Analysis (NTA)

The size distribution and concentration of N-ELNs were determined using a nanoparticle tracking analyzer (Particle Metrix, ZetaVIEW, Herrsching, Germany). The instrument was calibrated with 100 nm polystyrene beads diluted appropriately before analysis. Purified EVs were diluted in PBS to obtain the optimal particle concentration for analysis. NTA was conducted at room temperature using a 520 nm laser and the standard “EV_520” protocol, with vesicle movement recorded automatically at numerous positions. To calculate the particle size distribution and concentration, the resulting data were processed using the instrument software (version 8.05.14 SP7).

### 4.5. Transmission Electron Microscopy (TEM)

The morphology of N-ELNs was characterized using a transmission electron microscope (JEOL, JEOL JEM-1400, Tokyo, Japan). A 10 μL aliquot of purified EVs was adsorbed onto a Formvar/carbon-coated copper grid for 20 min. Then, the grid was subjected to negative staining with 2% phosphotungstic acid (pH 7.0) for 2 min and air-dried. Micrographs were acquired at an accelerating voltage of 80 kV.

### 4.6. Cellular Uptake of N-ELNs

N-ELN uptake by cells was assessed using the ExoGlow™-Membrane EV Labeling Kit (System Biosciences, EXOGM600A-1, Palo Alto, CA, USA). In brief, 100 μg of N-ELNs were incubated with a fluorescent labeling reagent for 30 min in the dark at room temperature. Excess unincorporated dye was removed using a 30 kDa molecular weight cut-off (MWCO) ultrafiltration unit via centrifugation at 10,000× *g* for 30 min. The labeled N-ELNs were then washed with PBS and resuspended to volume. To control for non-specific labeling, a negative control was prepared by processing an equivalent volume of PBS with the same fluorescent dye under the same purification and washing conditions. B16-F10 cells were incubated with either the labeled N-ELNs (50 μg/mL) or the control dye solution under identical conditions for 24 h. The cellular uptake of fluorescently labeled N-ELNs was visualized and captured using a confocal laser scanning microscope.

### 4.7. Cell Viability Assay

The cytocompatibility of N-ELNs toward B16-F10 melanoma cells and HFF-1 human foreskin fibroblasts was assessed using the Cell Counting Kit-8 (CCK-8) assay (Beyotime, C0037, Haimen, China). Cells in the exponential growth phase were collected and seeded into 96-well culture plates at a density of 5.0 × 10^3^ cells per well in 100 μL of complete medium. After a 24 h incubation to permit cell adhesion and stabilization, the medium was replaced with fresh medium supplemented with varying concentrations of N-ELNs (2.5, 5, 10, 20, and 40 μg/mL), with the 0 μg/mL group serving as the untreated control. Cells were then exposed to N-ELNs for 24 or 48 h to evaluate time-dependent responses.

After the designated incubation periods, 10 μL of CCK-8 reagent was added to each well. Plates were incubated for 2 h to allow chromogenic development. The absorbance was measured at 450 nm using a microplate reader. Cell viability was expressed as a percentage relative to the untreated control group (100%).

### 4.8. Wound Healing Assay

B16-F10 cells were plated in 6-well culture plates at a density of 4.0 × 10^5^ cells/well and incubated at 37 °C for 12 h to allow attachment. Upon reaching 80–100% confluence, a linear scratch was generated using a sterile 200 µL pipette tip. Detached cells were removed by washing the wells three times with PBS. Cells were then cultured in N-ELN-supplemented medium at concentrations of 0, 10, and 20 µg/mL. Images of the wound area were captured using a light microscope at 0, 24, and 48 h. Wound closure was quantified using ImageJ software, and migration rates were calculated using the following formula:

$$\mathrm{wound}\;\mathrm{healing}\;\mathrm{rate}=\frac{\mathrm{wound}\;\mathrm{area}\;(0\;h)-\mathrm{wound}\;\mathrm{area}\;(24\;h)}{\mathrm{wound}\;\mathrm{area}\;(0\;h)}$$

### 4.9. Effect of N-ELNs on Melanin Content

B16-F10 cells were seeded into 96-well plates at a density of 1.5 × 10^4^ cells per well and incubated for 12 h. The experimental design included the following groups: an untreated control group, a model group stimulated with 2 μmol/L α-melanocyte-stimulating hormone (α-MSH), a positive control group treated with 1 mM arbutin following α-MSH induction, and experimental groups co-treated with α-MSH and varying concentrations of N-ELNs. After treatment, the plates were incubated in a CO_2_ incubator for 48 h. Culture supernatants were collected and centrifuged to remove cellular debris, and 150 μL of each supernatant was transferred to a fresh 96-well plate. Melanin content was quantified by measuring absorbance at 475 nm using a microplate reader.

### 4.10. Cellular Tyrosinase Activity Assay

The B16-F10 cells were treated as described in Section 4.9. Following a 48 h incubation, cells were washed with PBS and lysed using 1% Triton X-100 (Sigma-Aldrich, St. Louis, MO, USA). The lysates were centrifuged at 12,000× *g* for 15 min at 4 °C to obtain the supernatant. Subsequently, 80 μL of the supernatant was mixed with 20 μL of L-3,4-dihydroxyphenylalanine (L-DOPA; 2 mg/mL; Sigma-Aldrich, D9628, St. Louis, MO, USA) and incubated at 37 °C for 1 h. Tyrosinase activity was determined by measuring the absorbance at 475 nm.

### 4.11. Measurement of N-ELN Effects on Melanogenesis-Related Gene Expression

B16-F10 cells were seeded at 4 × 10^4^ cells per well into 24-well plates and cultured for 12 h. The cells were then treated with N-ELNs at varying concentrations. Total RNA was isolated using TRIzol reagent (Sigma-Aldrich, T3809, St. Louis, MO, USA), with RNA collected at 8 h for *Mitf* analysis and at 48 h for *Tyr* and *Tyrp1* analysis. RNA integrity and purity were verified, and complementary deoxyribonucleic acid was synthesized using a Moloney murine leukemia virus reverse transcription kit (Vazyme, R021-01, Nanjing, China). Gene expression levels were quantified by real-time quantitative polymerase chain reaction (RT-qPCR) using GAPDH as the endogenous reference gene [54]. Primer sequences are provided in Table 1.

### 4.12. Statistical Analysis

Statistical analyses were performed using GraphPad Prism (v8.0.2; GraphPad Software, Inc., San Diego, CA, USA). Comparisons between two groups were evaluated using Student’s *t*-test. Differences among multiple groups were analyzed by one-way analysis of variance (ANOVA), followed by Tukey’s post hoc test. Statistical significance was defined as * *p* < 0.05, ** *p* < 0.01, and *** *p* < 0.001.

## 5. Conclusions

In summary, N-ELNs were successfully isolated and systematically characterized from *N. oculata*, displaying general physicochemical features consistent with extracellular nanovesicles. N-ELNs exhibited no detectable cytotoxic effects on B16-F10 melanoma cells or normal human dermal fibroblasts (HFF-1), nor did they adversely affect cellular migratory capability, thereby demonstrating favorable biocompatibility and biological safety. Furthermore, N-ELNs significantly suppressed α-MSH-induced melanogenesis in a dose-dependent manner, as evidenced by reduced melanin content and tyrosinase production. Mechanistic investigations indicated that the whitening effects of N-ELNs are mediated through the downregulation of the master transcription factor *Mitf* and its downstream melanogenic genes *Tyr* and *Tyrp1*, leading to the inhibition of tyrosinase activity and subsequent attenuation of melanin synthesis. Collectively, these findings underscore the dual potential of N-ELNs as intrinsic bioactive agents and promising nanocarrier systems. This study provides a scientific foundation for developing safe and effective cosmeceutical formulations and offers new perspectives for the high-value use of microalgal resources.

## Figures and Tables

**Table 1:** Primer sequences for RT-qPCR analysis.

Gene Name	Forward Primer (5′-3′)	Reverse Primer (5′-3′)
*Mitf*	GGGAAATGCTAGAATACAGCTACT	CTCCCCAGCTGGTTTTGGACA
*Tyr*	TCGGGATGAGAACTTCACTG	ACGTAAATGGTCCCTCTACGG
*Tyr1*	GCTGCAGGAGCCTCTTCTTC	AAGACGCTGCACTGCTGGTC
*GAPDH*	ACTCACGGAAATTCACGG	GACTCCACCACATACTGAGC

## Data Availability

The original contributions presented in this study are included in the article. Further inquiries can be directed to the corresponding authors.
